# The Use of Evidence-Informed Deliberative Processes for Health Insurance Benefit Package Revision in Iran

**DOI:** 10.34172/ijhpm.2022.6485

**Published:** 2022-03-02

**Authors:** Mojtaba Nouhi, Rob Baltussen, Seyed Sajad Razavi, Leon Bijlmakers, Moahmmad Ali Sahraian, Zahra Goudarzi, Parisa Farokhian, Jamaleddin Khedmati, Reza Jahangiri, Alireza Olyaeemanesh

**Affiliations:** ^1^National Institute for Health Research, Tehran University of Medical Sciences, Tehran, Iran.; ^2^Radboud University Medical Centre, Nijmegen, The Netherlands.; ^3^Mofid Children Hospital, Shahid Beheshti University of Medical Sciences, Tehran, Iran.; ^4^Multiple Sclerosis Research Center, Neuroscience Institute, Tehran University of Medical Sciences, Tehran, Iran.; ^5^Health Human Resources Research Center, Department of Health Economics, School of Management and Medical Informatics, Shiraz University of Medical Sciences, Shiraz, Iran.; ^6^High Council for Health Insurance, Ministry of Health and Medical Education, Tehran, Iran.; ^7^Pharmaceutical Management and Economics Research Center, Tehran University of Medical Sciences, Tehran, Iran.; ^8^Health Management and Economics Research Center, Iran University of Medical Sciences, Tehran, Iran.; ^9^Health Equity Research Center, Tehran University of Medical Sciences, Tehran, Iran.

**Keywords:** Universal Health Coverage, Iran, Multiple Sclerosis, Benefit Package, Deliberative Processes, Revision

## Abstract

**Background:** Iran considers the revision of its health insurance benefit package (HIBP) as a means to achieve universal health coverage (UHC). Yet, its decision-making process has been criticised for being weak in terms of accountability and transparency. This paper reports on the development and implementation of the HIBP revision in Iran in the period 2019-2021, employing evidence-informed deliberative processes (EDPs), a framework for benefit package design with the explicit aim of optimising the legitimacy of decision-making.

**Methods:** The High Council for Health Insurance (HCHI) is coordinating the HIBP revision: it planned the six steps of the EDP framework with support from World Health Organization (WHO) and Radboudumc in 2019, and conducted a pilot project on multiple sclerosis (MS) diagnosis and treatment in 2020.

**Results:** Implementation of the MS pilot project concerned the installation of advisory committees (involving some 60 stakeholders in supportive task forces, a technical working group [TWG] and a national advisory committee [NAC]), the selection of decision criteria (relating to quality of care, necessity, and sustainability), the inclusion of services for evaluation (nine in total), and the assessment and appraisal of these services.

**Conclusion:** Implementation of the priority setting process for MS diagnosis and treatment services has likely improved the legitimacy of decision-making by involving stakeholders who engaged in deliberation based on available evidence in a stepwise, transparent process. It is expected to improve the quality of care for MS patients as well as its financial accessibility, at a zero net budget impact. The pilot project has served to help Iran’s health system move faster toward UHC for a broader range of essential health services.

## Background

**Key Messages**

** Implications for policy makers**

- The revision of the Health Insurance Benefit Package (HIBP) based upon selected important conditions can provide better access to new services and improve universal health coverage (UHC) situation in Iran.
- The use of evidence-informed deliberative processes (EDPs) holds large potential to improve the legitimacy of HIBP revision in Iran, by involving stakeholders who engaged in deliberation based on available evidence in a stepwise, transparent process.
- Implementation of the priority setting in a pilot project on multiple sclerosis (MS) control informed a reallocation of resources, and is expected to improve the quality of care for patients as well as its financial accessibility, at a zero net budget impact.

** Implications for the public**

The huge share of the diagnostic and treatment services provided to patients by providers in Iran are in the Health Insurance Benefit Package (HIBP). The services of this package need to be revised regularly to include new services and remove low-value services. This paper presents a new approach to revise the HIBP with an emphasis on transparency and stakeholder involvement, in an effort to align decisions with public preferences and to optimize public support. The approach is successfully applied to the multiple sclerosis (MS) which led to the inclusion of four new services and revised coverage for five other services.

Like many other countries around the world, Iran strives for universal health coverage (UHC).^1-3^ Yet, the Iranian health system is characterised by a range of systemic inefficiencies, caused by a strong emphasis on curative procedures and specialist services, supplier-induced demand and a high consumption of low-value care.^4-6^ These all contribute to escalating healthcare expenditures.^7^

The Ministry of Health and Medical Education (MoHME) considers the revision of the health insurance benefit package (HIBP) as a top priority in its policy agenda, and as a means to ensure UHC and financial sustainability of the health system especially in the context of societal pressure to cover (expensive) emerging medical technologies.^5,8^ The HIBP constitutes the secondary and tertiary level services that social health insurance agencies in Iran offer. It is complementary to the governmental health package offered by primary level facilities which comprises vaccination, screening services and mother and childcare.

The HIBP decision-making process has been criticised for being weak in terms of accountability and transparency.^9^ Policy-makers in Iran, as elsewhere around the globe, are increasingly urged to organise such processes in a fair and legitimate manner, defined here as the reasonableness of decisions as perceived by stakeholders.^10,11^ This paper reports on the development and implementation of HIBP revision in Iran in the period 2019-2021, employing evidence-informed deliberative processes (EDPs). The EDP framework is a practical and stepwise tool for priority setting, rooted in health technology assessment, with the explicit aim to optimize the legitimacy of benefit package decisions.^12-15^ In this framework, the concept of legitimacy is translated into four elements – stakeholder involvement, ideally operationalised through stakeholder participation with deliberation; evidence-informed evaluation; transparency; and appeal. The practical guidance on EDPs provides recommendations on how these elements can be implemented in each step of the decision-making process of HIBP design. Several other frameworks for priority setting are available^10,16-18^ to which the EDP framework can be considered as complementary because of its explicit focus on stakeholder participation. The present approach follows up on previous initiatives on HIBP design in Iran.^19-30^

The main aim of HIBP revision in Iran is to facilitate the reallocation of resources from low-value to high-value services for a range of conditions within six selected disease areas (cancer, metabolic disorders, mental disorders, respiratory diseases, neurological disorders, and cardiovascular disorders), with the explicit objective to remain within a certain budget limit. HIBP revision is considered an ongoing activity in which various services targeting certain conditions in different disease areas are periodically evaluated. This paper describes the first evaluation of a set of services targeting multiple sclerosis (MS),^3^ as one of the common conditions in the neurological disorders’ disease area, carried out in 2020-2021.

 The paper starts with a description of the development of the general framework for HIBP revision in Iran, followed by the reporting on the MS pilot project. We conclude by drawing lessons from this pilot project for future HIBP revision in other disease areas.

## Methods

###  Development of General Framework for HIBP Revision

 HIBP design is the responsibility of the High Council for Health Insurance (HCHI), a body within the MoHME. The HCHI is composed of representatives of nine formal entities including the MoHME’s Curative Affairs Department, the Ministry of Cooperative, Labour, and Social Welfare, the Ministry of Finance and Economic affairs, the Medical Council, the Planning and Budget Organization, and three social health insurance agencies. The HCHI is chaired by the Minister of MoHME.

**Figure 1:**
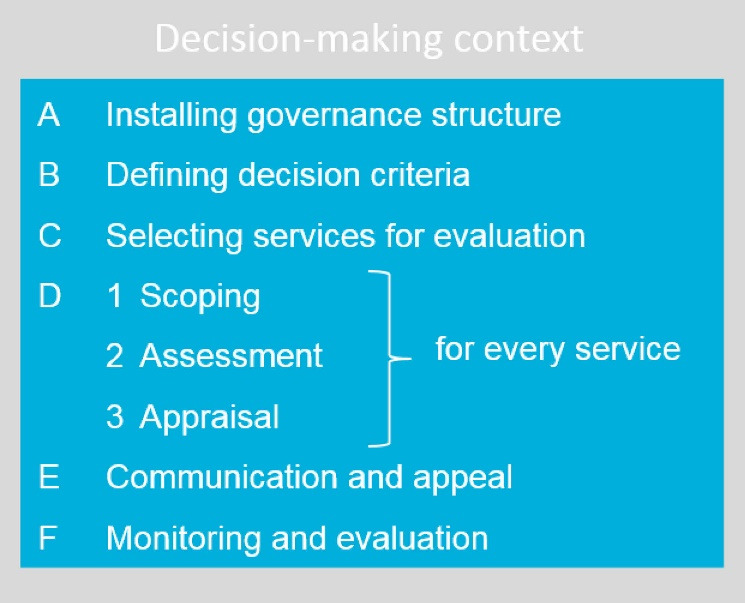
Practical Steps of the Evidence-Informed Deliberative Process for Health Insurance Benefit Package Revision.

HCHI is coordinating the HIBP revision with support from the World Health Organisation and Radboud university medical center (Radboudumc). The EDP framework was adapted and operationalised during several rounds of consultation with stakeholders in Iran (May 2019, February 2020) and received approval by the Steering Committee in April 2020. The adapted EDP framework is provided in Figure 1.

###  Step A: Installing a Governance Structure

**Figure 2:**
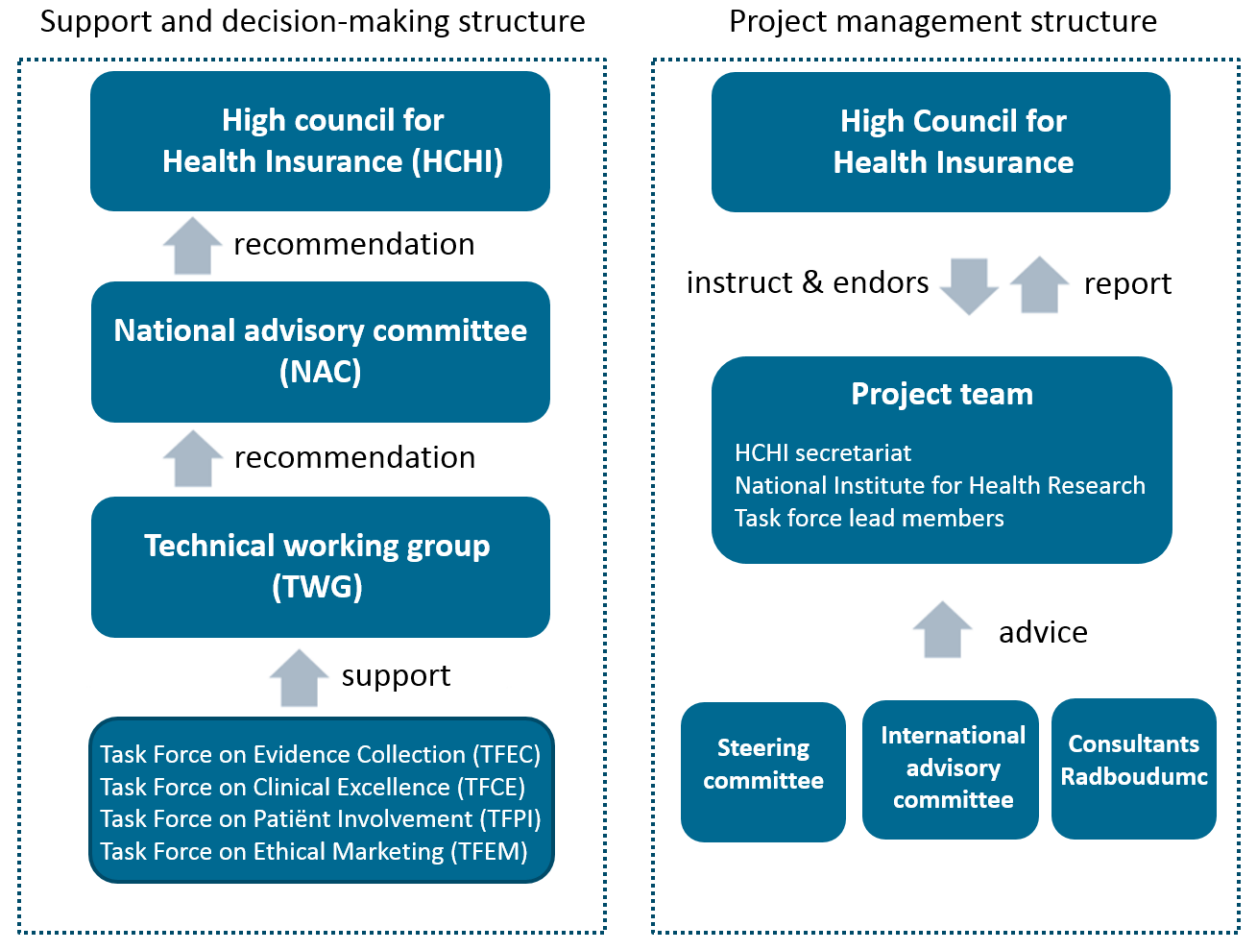
The Health Insurance Benefit Package Governance and Project Management Structure.

The HCHI designed a HIBP governance structure including the following bodies: Task forces, technical working groups (TWGs), a national advisory committee (NAC) and the HCHI. (Figure 2, left panel). Terms of reference were drafted and adopted for each body. Box 1 describes the composition and tasks of the governance bodies put in place as part of the governance structure that oversees the HIBP revision process. The project management structure consists of a Project team which takes care of the day-to-day coordination of the HIBP revision, and involves members of the HCHI secretariat, the National Institute for Health Research and Task Force lead members (Figure 2, right panel). The Project team receives guidance from the Steering committee, an International advisory board, and international consultants from Radboudumc.

**Box 1. Tasks and Composition of the Governance Bodies**

The first type of governance bodies are Task Forces. The TFCE is established per condition, each including some 10 members involving specific expertise on clinical and health service aspects on the condition under study. The TFCE prepares input to the TWG, ie, a mapping of services for the condition including service utilization patterns. The TFEC is responsible to collect the key evidence and needed utilization data on selected conditions in the prioritised six disease areas to the TFCE based on local and international databases. The TFEC also develops initial recommendations on service coverage to the TWGs (see below). The TFEC is a technical team including experts such as a clinical specialist, health economist, epidemiologist, pharmacologist, health system specialist, data analyst and a service provider. Depending on the condition, members can be added. The TFPI is responsible to involve patients or representatives of patients on the processes of coverage decision making and revision of HIBP. The TFEM is responsible for regulating the relationship between pharmaceutical companies, physicians and insurance agencies, and more specifically that marketing for pharmaceutical products. Whereas the TFCE is established per condition, the composition of TFEC, TFPI and TFEM is identical across conditions.

The second type of bodies are TWGs. TWGs are tasked with reviewing the technical aspects of the services as prepared by the Task Forces, and developing judgments on the relative priority of these services leading to coverage recommendations. TWGs are established per disease area, each including some 10-15 members, involving representatives of TFCE, and other relevant stakeholders such as medical professionals, patients, scientists, representatives from insurance companies, members of HCHI. The TWG is active in Scoping (step D1) and Appraisal (step D3) – these steps are described in more detail below. The TWG reports its recommendations to the NAC for endorsement.

The third body is the NAC whose mandate is to appraise services, based on the recommendations of the TWGs. The NAC includes 24 members including the Treatment Deputy and the Health Deputy of the MoHME, and representatives from the Iran Food and Drug Organization, the Medical Council, the Ministry of Economic Affairs and Finance, the Ministry of Cooperatives, Labour & Social Welfare, and the Planning and Budget Organization. The NAC is chaired by the Secretary of the HCHI or his/her deputy. Selected TWG members may attend NAC meetings to present their recommendations and supporting argumentation. The NAC is active in Appraisal (Step D3).

The fourth body is the HCHI. The NAC reports its recommendations to the HCHI for endorsement. The HCHI forwards the recommendations to the Minister of MoHME for adoption.

--------------------

Abbreviations: TFCE, Task Force on Clinical Excellence; TWG, technical working group; TFEC, Task Force on Evidence Collection; TFPI, Task Force on Patient Involvement; HIBP, Health Insurance Benefit Package; TFEM, Task Force on Ethical Marketing; NAC, national advisory committee; MoHME, Ministry of Health and Medical Education; HCHI, High Council for Health Insurance.

###  Step B: Defining Decision Criteria

**Figure 3:**
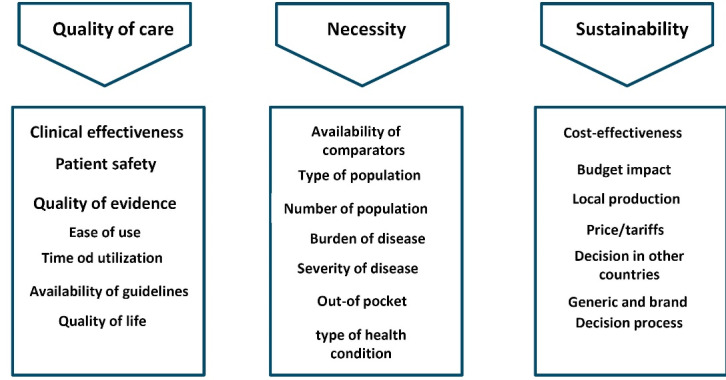
Decision Criteria for HIBP Design as Classified in Three Pillars. Based on Viyanchi et al,^28,29^ Mohammadi et al,^25^ Kabir et al,^24^ Nouhi,^30^ Dehnavieh et al.^20^ Abbreviation: HIBP, health insurance benefit package.

Each service needs to be evaluated according to a set of decision criteria reflecting the key aspects that determine its value. Previous surveys and consultations in Iran identified the criteria severity of disease, treatment effectiveness, treatment safety, patient satisfaction, cost of diagnosis and treatment, budget impact, local production of required technology (instruments, medication), out-of-pocket expenses, availability of an alternative form of treatment, and size of the population affected by the disease/condition. These are classified in three broad pillars of main criteria: ‘quality of care,’ ‘necessity’ and ‘sustainability’ (Figure 3).^31^ This classification is retained in the present approach. The operationalisation of these criteria took place in the MS pilot project (see below).

###  Step C: Selecting Services 

 This step involves two activities. Firstly, HCHI selected six main disease areas and will subsequently select conditions within each of these areas (if known, the latter is indicated between brackets): neurological disorders (MS), metabolic disorders (diabetes mellitus), cancers, cardiovascular diseases, respiratory diseases, and mental health. The selection is based on the burden of disease and financial risk in accessing services. Secondly, the Task Force on Clinical Excellence (TFCE) identifies all existing and new promising services for these conditions. Services are classified on the basis of their phasing in managing the condition, ie, diagnosis, treatment and follow-up, providing a good overview of which services are interchangeable and which resources can in principle be reallocated. In addition, the Task Force on Evidence Collection (TFEC) collects basic evidence for these services in terms of their use, coverage by HIBP and costs.

###  Step D1: Scoping 

 The TWGs define the scope of analysis on the basis of information provided by the TFEC, ie, the evaluation questions that are addressed in the subsequent assessment and appraisal steps. This involves the following elements:

- Which services should be evaluated? For reasons of capacity, it is virtually impossible to evaluate and review all possible services targeting a condition. The focus is therefore on services that have the highest budget impact or are suspected of having limited effectiveness in combination with high usage levels.
- Which decisions need to be made? These may include decisions to in-or exclude, conditional coverage, utilization and/or price reduction, or service modification.
- Which evidence needs to be collected in order to respond to these questions?

###  Step D2: Assessment 

 The assessment of services is carried out by the TFEC and relates to the construction of the evidence base on which the services are evaluated, ie, the performance of services on all decision criteria. Given the scope of analysis (evaluating several conditions for each of six disease areas), we decided to use descriptive data on criteria such as costs, health impact and utilization patterns, and not to use dynamic models. Sources include scientific literature, real world data and expert opinion. All evidence is summarised in evidence sheets, using icons for each criterion and professional lay-out, for presentation to the TWG.

 On the basis of provided evidence and knowledge of its members, the TFEC scores the performance of services using a classification of stars: high value (three stars), medium value (two stars) or low value (one star) for each pillar of criteria. The TFEC also provides an initial recommendation on the service to the TWG.

###  Step D3: Appraisal

 In the appraisal step, the TWGs develop recommendations on the in- or exclusion of certain services for a certain condition. They do this with explicit reference to evidence on the performance of each service according to three pillars of criteria: ‘quality,’ ‘necessity’ and ‘sustainability’ – as provided by the TFEC. This appraisal is straightforward if a particular service performs well on all criteria, ie, if it is of high quality (eg, high clinical effectiveness), high necessity (eg, no alternative service) AND high sustainability (eg, low costs): the recommendation will then be to include the service. Likewise, if a services scores poorly on all three criteria, it will be recommended for exclusion. However, the appraisal is more challenging if a service scores well on only one or two criteria but poor on one or two others – in that case TWG members need to balance and judge the importance of the three pillars of criteria. The TWGs evaluate all services targeting a condition with regards to the current level of spending on that condition. The latter confronts the TWG members with the need to disinvest in some services if they propose to expand others.

 The TWG reports its recommendations to the NAC. The NAC makes its own judgement and reports its recommendations to the HCHI which formally approves the recommendations.

###  Step E: Communication and Appeal

 For reasons of transparency and legitimacy, it is important to clearly describe the general decision-making process, including the decision criteria deemed relevant, in a publicly available document. In addition, the eventual decisions on the in- and exclusion of services should also be made publicly available, including the argumentation and evidence used.

 An appeal mechanism needs to be installed that requires the advisory committee to review a certain decision once new data or new insights become available. The HCHI is yet to establish the publicity and appeal mechanisms.

###  Step F. Monitoring and Evaluation

 After the NAC and HCHI have endorsed the recommendations, the selected services will be provided and the excluded services phased out. HIBP implementation should be evaluated to ensure that the package is adhered to and to safeguard service quality. The HCHI is yet to establish the monitoring and evaluation procedures.

## Results

###  Implementation of HIBP Revision: The Pilot Project on Multiple Sclerosis 

 The Project team selected MS as pilot project because of the availability of relevant data; the limited number of patients (<100 000) making the implementation of a revised service package relatively easy; the urgent financial challenges related to new and expensive medication to treat MS patients; and the expressed interest of neurologists to be involved in the prioritisation process.

The MS pilot project lasted from May 2020 to March 2021 and involved online workshops on the EDP framework (Figure 1) and its implementation, organised by Radboudumc with Project team participants from HCHI (July-September 2020). The project followed the steps of the EDP framework (Figure 1). Manuscript authors were engaged in the implementation of the MS pilot project, and results are reported on the basis of participatory observation.

*Step A: *We established the TFCE on MS with 11 members, including physicians and clinical specialists on neurology, a health economist, an epidemiologist, and a primary healthcare expert. The TWG on Neurology comprised 25 members, including representatives of MoHME, Ministry of Welfare, Ministry of Economic Affairs, three social health insurance agencies, IFDA, the TFEC, TFCE, the Planning and Budget Organization, MS Patient Support Union (the Iranian MS patient interest group), Medical council, and five neurologists from different provinces. The Task Force on Patient Involvement (TFPI) informed representatives of the MS Patient Support Union on the HIBP process and possible ways to get involved. Three representatives of the MS Patient Support Union participated in the TWG, and one representative participated in the NAC.

*Step B: *The MS pilot project employed the generic decision criteria as described above. The operationalization of these criteria took place during evidence collection in step C (see Supplementary file 1) and step D2 (see Supplementary files 2 and 3).

*Step C: *To inform the selection of services for evaluation by the TWG, the the TFEC developed an evidence base on MS. This included:

- A mapping of presently covered MS services divided in five clusters: diagnosis and risk stratification (including laboratory diagnostic and imaging services); clinical management; relapse management; symptom management (to treat side-effects such as fatigue and depression); and patient follow-up. The list of services per cluster is provided in Supplementary file 1.
- The TFE collected evidence for each of these services in terms of their use, coverage by HIBP and costs. It turned out that the clinical management of MS accounted for more than 83% of the total MS budget in 2019.

*Step D1: *The TWG held two scoping meetings in November 2020 to identify services for closer evaluation, possible forthcoming decisions and required additional evidence in terms of quality (ie, clinical impacts), necessity (ie, financial risk protection) and sustainability (ie, budget impact) (Table 1). The TWG did not require evidence on other decision criteria.

**Table 1:** The TFEC Scores of Evaluated Multiple Sclerosis Services on Three Pillars of Criteria and Initial TWG Recommendations^a^

**Service**	**TFEC Initial Recommendation/TWG Proposed Decision **	**Quality of Care**	**Necessity**	**Sustainability**
Anti-NMO antibody	Inclusion	Positive impact**	Positive impact*	Negative impact*
CSF analysis	Inclusion	Positive impact**	Positive impact*	Negative impact*
Alemtuzumab	Inclusion	Positive impact*	Positive impact***	Negative impact***
Ocrelizumab	Inclusion	Positive impact**	Positive impact***	Negative impact***
Extavia	Exclusion	No difference	Negative impact*	Positive impact*
Betaferon	Exclusion	No difference	Negative impact*	Positive impact*
Rebif	Exclusion	No difference	Negative impact*	Positive impact*
Avonex	Exclusion	No difference	Negative impact*	Positive impact*
Cinnovex	Conditional coverage	Negative impact*	Negative impact*	Positive impact***

Abbreviations: TWG, technical working group; TFEC, Task Force on Evidence Collection; CSF, cerebrospinal fluid; NMO, neuromyelitis optica. ^a^The stars reflect the magnitude of impact (small, medium and large value, indicated as 1, 2 and 3 stars respectively).

*Step D2:* The TFEC collected the additional evidence (see Supplementary file 1, Table S1 for a full overview) and provided an assessment of the performance of the services in terms of their direction (positive, negative, or no difference) and magnitude of impact (small, medium and large impact, indicated as 1, 2 and 3 stars respectively). In addition, the TFEC provided initial recommendations which were for all services similar to the TWG proposed decisions (Table 1). As an example, both TWG and the TFEC proposed to include ocrelizumab in the HIBP as a substitute for natalizumab. In comparison to the latter medicine, ocrelizumab was considered of higher quality (safer, with lower relapse rates, and requiring fewer injections); it scored better on the necessity criterion (requiring fewer inpatient procedures, hence involving less co-payment), but worse on sustainability (being more costly).

*Step D3: *The TWG appraised the services on the basis of the presented evidence in a third workshop in March 2021 and provided their final recommendations (Table 2).

**Table 2:** Recommendation for Technical Working Group on Multiple Sclerosis

**Service**	**Final Recommendation**	**Reallocation of Resources**
Anti-NMO antibody	Included only when based on clinical guidelines	(306 905)
CSF analysis	Included only when based on clinical guidelines	(43 000)
Alemtuzumab	Included with using risk sharing agreement	(1 044 286)
Ocrelizumab	Included with using price-volume agreement	(6 309 524)
Extavia	Using internal reference pricing	147 882
Betaferon	Using internal reference pricing	202 381
Rebif	Using internal reference pricing	37 857
Avonex	Using internal reference pricing	725 000
Cinnovex	Using internal reference pricing	6 590 595

Abbreviations: NMO, neuromyelitis optica; CSF, cerebrospinal fluid.

 Overall, these final recommendations were more nuanced and conditional of nature as compared to the initially proposed inclusion/exclusion recommendations in the scoping step. The reason is that the include/exclude dichotomy made it difficult for stakeholders to reach consensus. As an alternative, conditional coverage options were introduced to facilitate the discussion and reduce the financial and clinical risk of decisions. Two services (anti-NMO [neuromyelitis optica] antibody and CSF [cerebrospinal fluid] analysis) were newly proposed for reimbursement on the condition that their use is indicated based on available clinical guidelines. One medicine, alemtuzumab, was newly proposed for reimbursement on the condition that it would meet effectiveness and safety requirements. Another medicine, ocrelizumab, was newly proposed for reimbursement, provided that manufacturers offer it at a lower cost in view of the expected rise in consumption once social health insurance agencies start reimbursing it. Five medicines (Extavia, Betaferon, Avonex, Rebif, and Cinnovex) were already being reimbursed but the TWG recommended stricter coverage policies based on reference pricing, ie, only up to the price level of a cheaper alternative product (Actovex).

The total annual extra costs were estimated at US$7 703 715 and the total annual savings at US$7 703 715. This means that the total set of recommendations come at a zero net cost. The zero net costs is not a coincidence as the price arrangements were set to meet this goal. Details are provided in Table 2.

 The TWG sent these recommendations to the NAC. Upon discussion, the NAC endorsed the recommendations on March 9, 2021. The HCHI approved the revisions to the MS package on March 16, 2021.

## Discussion

 This paper has described the development of a standard framework for HIBP revision in Iran, through the use of EDPs, and its piloting. The pilot project on MS convincingly illustrates the feasibility of the approach, with the final decisions on the conditional coverage of nine MS services within a time frame of 10 months.

 The use of EDPs has likely improved the legitimacy of the HIBP decision process in Iran in three ways. First, the process involved some 60 stakeholders through TFCE, TWG and NAC membership, thereby drawing broad support for the decisions that were taken. Second, the process was evidence-informed; the discussions not only relied on formal evidence but also used stakeholders’ and experts’ judgements where relevant and necessary. These brought in valuable expertise on especially the practical implementation of the MS service package, which added to the credibility of the decision-making process as a whole. Third, the use of EDPs led to more transparency of decision making because many stakeholders were involved and are now fully informed.

 The revised HIBP of MS services can also be expected to lead to a better quality of care for MS patients. The benefit package now includes medicines with high levels of safety and effectiveness, such as ocrelizumab and alemtuzumab which replace natalizumab. Also the reimbursement of CSF is likely to improve the differential diagnosis of MS. The revised HIBP may also improve the financial accessibility of services, as price arrangements (price volume agreements and reference pricing) make them more affordable for patients. In terms of sustainability, the MS HIBP revision has a zero net budget impact: the exclusion of low-value services compensates for the inclusion of high-value services. However, these are provisional estimates and the actual future budget impact will be dependent on further developments, such as possible changes in treatment protocols or commercial agreements between insurance agencies and pharmaceutical companies. This indicates the need to install adequate monitoring and evaluation system, to assess the impact of the HIBP revision over time eg, to judge whether estimated cost savings will indeed be realized.

Several issues emerged during EDP implementation in the MS pilot project, each requiring special attention in future HIBP revisions. Firstly, participation in the advisory committees could be improved in terms of inclusive member recruitment, in particular representation of the general public. Yet, MS patient representatives formally participated in the TWG and NAC which can be considered as an important element of legitimacy.^15^ Future monitoring and evaluation activities should demonstrate whether this participation has also been meaningful, and as to whether patient – as well as other stakeholders – find that their perspectives were taken into account.

Second, the project team employed three main *decision criteria*, each divided into several sub-criteria. Several of them were not sufficiently operationalized. As a consequence, the main criterion ‘sustainability’ was largely based on budget impact analysis, the criterion ‘quality’ on clinical benefit and the criterion ‘necessity’ on financial risk protection. The evaluation of services in terms of other sub-criteria, such as size of the targeted population, cost-effectiveness (for diagnostic services), disease severity, risk of death for patient, and patients’ clinical need, were based entirely on expert judgements

Fourth, the MS pilot project shows it was possible to reach consensus among stakeholders in the* appraisal phase*. One of the enablers was the explicit budget constraint that was given: it may have served as an incentive for social health insurance agencies to accept some of the proposed revisions that implied less expenditure. Another enabler may have been the use of conditional coverage options, reducing and/or sharing the financial risk among stakeholders. These aspects are worth including in upcoming HIBP revisions for other conditions, also in other disease areas.

Fifth, the project improved efficiency within MS control but did not address broader efficiency issues across diseases, which may require certain reallocations of resources, for example from relatively low-value MS services to high-value diabetes mellitus services. For the latter, the NAC should consider benefit package revisions involving multiple diseases at the same time. Sixth, on *communication and appeal*, the HCHI is publishing decision and materials on its website with public access for all citizens. However, it is yet to reach a more comprehensive processes for publicity and appeal mechanism.

 Finally, the outbreak of coronavirus disease 2019 (COVID-19) and the subsequent restrictive measures have formed a limitation to the EDP process, in that they have affected stakeholder participation and the quality of the deliberations. This has likely affected stakeholder participation and the quality of the decision-making process.

## Conclusion

 Implementation of the priority setting process for MS diagnosis and treatment services has likely improved the legitimacy of decision-making by involving stakeholders who engaged in deliberation based on available evidence in a stepwise, transparent process. It is expected to improve the quality of care for MS patients as well as its financial accessibility, at a zero net budget impact. The pilot project has served to help Iran’s health system move faster toward UHC for a broader range of essential health services.

## Acknowledgements

 We thank to all people who participated in TWGs and NAC meetings.

## Ethical issues

 This project was approve by ethical committee at Tehran University of Medical Sciences (Approval ID: IR.TUMS.NIHR.REC.1399.021).

## Competing interests

 Authors declare that they have no competing interests.

## Authors’ contributions

 Conception and design: RB and MN. Acquisition of data: JK and SSR. Analysis and interpretation of data: MN and MAS. Drafting of the manuscript: RB and MN. Critical revision of the manuscript for important intellectual content: RB and MN. Statistical analysis: MN, RJ, PF, and ZG. Obtaining funding: RB. Administrative, technical, or material support: MN. Supervision: RB and MN.

## Funding

 This project was funded by World Health Organization (WHO) Country Office in Iran (Islamic Republic of).

## Supplementary files


Supplementary file 1. Mapping of Presently Covered MS Services in Five Clusters.

Supplementary file 2. Evidence on Multiple Sclerosis and Available Services for Cluster 1 and 2 (as Collected by TFEC in Step C).

Supplementary file 3. Evidence on Multiple Sclerosis and Available Services for Cluster 3-5 (as Collected by TFEC in Step C).
