# Supplementary material for: The Use of Evidence-Informed Deliberative Processes for Health Insurance Benefit Package Revision in Iran
Source: Int J Health Policy Manag. 2022 Mar 2;11(11):2719–26. doi: 10.34172/ijhpm.2022.6485 (PMC9818091; doi:10.34172/ijhpm.2022.6485)
Supplement: Supplementary file 1 — Mapping of Presently Covered MS Services in Five Clusters. [file ijhpm-11-2719-s001.pdf]

Article title: Journal name: International Journal of Health Policy and Management (IJHPM)
Authors' information: Mojtaba Nouhi[1], Rob Baltussen[2]*, Seyed Sajad Razavi[3], Leon Bijlmakers[2], Moahmmad Ali Sahraian[4], Zahra Goudarzi[5], Parisa Farokhian[6], Jamaledding Khedmati[7], Reza Jahangiri[8], Alireza Olyaeemanesh[9]*

[1]National Institute for Health Research, Tehran University of Medical Sciences,Tehran, Iran.

[2]Radboud University Medical Centre, Nijmegen, The Netherlands.

[3]Mofid Children Hospital, Shahid Beheshti University of Medical Sciences, Tehran, Iran.

[4]Multiple Sclerosis Research Center, Neuroscience Institute, Tehran University of Medical Sciences, Tehran, Iran.

[5]Health Human Resources Research Center, Department of Health Economics, School of Management and Medical Informatics, Shiraz University of Medical Sciences, Shiraz, Iran.

[6]High Council for Health Insurance, Ministry of Health and Medical Education, Tehran, Iran.

[7]Pharmaceutical Management and Economics Research Center, Tehran University of Medical Sciences, Tehran, Iran.

[8]Health Management and Economics Research Center, Iran University of Medical Sciences, Tehran, Iran.

[9]Health Equity Research Center, Tehran University of Medical Sciences, Tehran, Iran.

(*Corresponding author: Email: rob.baltussen@radboudumc.nl & arolyaee@gmail.com)

Supplementary file 1. Mapping of Presently Covered MS Services in Five Clusters

Diagnosis and risk stratification

Imaging services:	Brain/cervical MRI contrast; CT scan
Lab tests:	Complete Blood Count (CBC), Alanine aminotransferase (ALT), Aspartate aminotransferase (AST), blood urea nitrogen (BUN), Creatinine, Thyroid-stimulating hormone (TSH), erythrocyte sedimentation rate (ESR), c-reactive protein test (CRP), antinuclear antibody (ANA), 25-Hydroxy Vitamin D3, Serum Level B 12
Services without HIBP coverage:	Anti NeuroMyelitis Optica (NMO) antibody, Cerebrospinal Fluid (CSF) analysis

Clinical management

Medicine:	Teriflunomide, Interferons (including medicines with different brands such as Rebif, Extavia, Avonnex, Betaferon and biosimilars such as Actovex and cinnovex), Dimethyl fumarate, Rituximab, Fingolimod, Glatiramer acetate, Mitoxantrone, Natalizumab
Services without HIBP coverage:	Alemtuzumab, Ocrelizumab, Cladribine, Siponimod

Relapse management

Services:	Steroid pulse therapy, Plasma exchange, Intravenous Immunoglobulin (IVIG)
Services without HIBP coverage:	not reported.

Symptom Management

For bladder dysfunction:	Oxybutynin, Tolterodine, Solifenacin, Botulinum toxin injection
For bowel dysfunction:	Consultation to change dietary to increase fluid and fiber intake, laxatives and enemassical examination
For fatigue:	Modafinil, Dextroamphetamine-amphetamine , Methylphenidate, Amantadine

For depression:	Selective serotonin reuptake inhibitor (SSRI), Serotonin–norepinephrine reuptake inhibitor (SNRI), Tricyclic antidepressant (TCA)
For spasticity:	Baclofen, Tizanidine, Butolinum toxin, Dantrolen, Benzodiazepine
For gaint impairment:	Physical therapy, dalfampridine
Services without HIBP coverage:	not reported.

Follow-up

Imaging services:	Every 3-6 month brain/spinal MRI ± contrast
Lab tests:	CBC , ALT , AST , BUN , Cr , TSH , ESR , CRP , ANA , 25 OH ViT D3 , Serum Level B 12
Services without HIBP coverage:	not reported.

* This overview only lists the most important services in terms of frequency of usage

Summary of performance of selected MS services on quality of care, necessity and sustainability, collected by TFEC in step D2

Table S1: Performance of selected MS services collected by TFEC in step D2.

Service	Quality<br>(clinical impact: effectiveness and safety)	Necessity<br>(OOP and alternative availability)	Sustainability<br>(Budget impact)
Ocrelizumab	Ocrelizumab enjoys a satisfactory level of effectiveness compared to Rituximab and it has much better clinical outcomes in comparison with Natalizumab.<br>Ocrelizumab has high level of safety in RRMS patients compared with all DMTs. It is also more convenient in terms of fewer number of injections.<br>Level of evidence: scientific studies (meta-analysis on clinical trials) and patients' opinion	Although there are other alternative medicines such as rituximab and Natalizumab in HIBP, OOP would exceed patient affordability.<br>Level of evidence: real world evidence (secretariat for HCHI)	Budget impact would be too much for social health insurance agencies.<br>Coverage of this medicine should be done carefully through a conditional coverage agreement – this would allow management of budget impact in short and long term.<br>Level of evidence: real world evidence (secretariat for HCHI)
CSF analysis	This test helps to differentiate between MS from other similar diseases. The diagnosis power is effective in prognosis of the disease progression in patients in CIS condition .No severe complications have been reported while using this service.	MRI and clinical examination are the conventional techniques to diagnose MS. However, suspected patients may need CSF test. OOP for this service is not unbearable.	The budget impact burden arising from access to this service is acceptable and will not jeopardize the long-term sustainability of social health insurance agencies.

Table S1: Performance of selected MS services collected by TFEC in step D2.

Service	Quality<br>(clinical impact: effectiveness and safety)	Necessity<br>(OOP and alternative availability)	Sustainability<br>(Budget impact)
	Level of evidence: scientific studies (clinical trials)	Level of evidence: real world evidence (secretariat for HCHI)	Level of evidence: real world evidence (secretariat for HCHI)
Alemtuzumab	Alemtuzumab is clinically of high effectiveness but relative not so satisfactory level of safety.<br>FDA reports Alemtuzumab may cause ischemic and haemorrhagic stroke and cervicocephalic arterial dissection, rare but serious adverse effects.<br>Level of evidence: scientific studies (meta-analysis on clinical trials) and real world evidence for safety warning	A small number of MS patients (RRMS and PPMS) who responded poorly to DMTs like Natalizumab can be the target population.<br>OOP is much beyond the patients' affordability.<br>Level of evidence: real world evidence (secretariat for HCHI)	The price of the medicine is too high and exerts a huge budget impact on the social health insurance agencies. Hence, it is recommended that the insurance coverage for this medicine be specified, transparently and meticulously observing the standard under supervision of the national MS committee.<br>Level of evidence: real world evidence (secretariat for HCHI)
Anti NMO antibody	The anti-NMO antibody diagnostic test is effective as a differential diagnosis of Devic from MS.	MRI and clinical examination are the two techniques in diagnosis of MS. However, for a differential diagnosis of MS from Devic, anti -NMO antibody is	The budget impact arising from the the service is acceptable and it will not suffer long-term sustainability of social health insurance agencies.

Table S1: Performance of selected MS services collected by TFEC in step D2.

Service	Quality<br>(clinical impact: effectiveness and safety)	Necessity<br>(OOP and alternative availability)	Sustainability<br>(Budget impact)
	No complex complications of high probability have been reported while using this service.<br>Level of evidence: scientific studies (observational studies)	required. OOP for this service is affordable.<br>Level of evidence: real world evidence (secretariat for HCHI)	Level of evidence: real world evidence (secretariat for HCHI)
Avonex	Given the reports and the published articles, the exclusion of the brand Given the reports and the published articles, the exclusion of Avonex does not negatively impact clinical outcomes of the patients.<br>Level of evidence: scientific studies (observational studies) and clinical experts' opinion	There are good alternative medicines for Avonex in HIBP. Few small share of patients who used this interferon received Avonex.<br>Clinical experts indicate that those patients could shift to the biosimilars without occurring clinical hazard. If they shift, OOP will decrease.<br>Level of evidence: real world evidence ((secretariat for HCHI) And clinical experts' opinion	If the patients shift to biosimilar of Interferon Beta B-1 , a total of 725,000 US$ estimated will be saved annually.<br>Level of evidence: real world evidence (secretariat for HCHI and Social health insurance agencies)

Table S1: Performance of selected MS services collected by TFEC in step D2.

Service	Quality<br>(clinical impact: effectiveness and safety)	Necessity<br>(OOP and alternative availability)	Sustainability<br>(Budget impact)
Betaferon	Like Avonex:<br>Given the reports and the published articles, the exclusion of Betaferon does not negatively impact clinical outcomes of the patients.<br>Level of evidence: clinical experts' opinion	Like Avonex:<br>Few small share of patients who used this interferon received Betaferon.<br>Clinical experts indicate that those patients could shift to the biosimilars without occurring clinical hazard. If they shift, OOP will decrease.<br>Level of evidence: real world evidence ((secretariat for HCHI)<br>And clinical experts' opinion	Estimated saving for social health insurance agencies: 202,381 US$ annually<br>Level of evidence: real world evidence (secretariat for HCHI and Social health insurance agencies)
Extavia	Like Betaferon:<br>Given the reports and the published articles, the exclusion of Extavia does not negatively impact clinical outcomes of the patients.<br>Level of evidence: clinical experts' opinion	A few small share of patients who used this interferon received Extavia.<br>Clinical experts indicate that those patients could shift to the biosimilars without occurring	Estimated saving for social health insurance agencies: 37,857 US$ annually<br>Level of evidence: real world evidence (secretariat for HCHI and Social health insurance agencies)

Table S1: Performance of selected MS services collected by TFEC in step D2.

Service	Quality<br>(clinical impact: effectiveness and safety)	Necessity<br>(OOP and alternative availability)	Sustainability<br>(Budget impact)
		clinical hazard. If they shift, OOP will decrease.<br>Level of evidence: real world evidence ((secretariat for HCHI)<br>And clinical experts' opinion	
Rebif	Like Betaferon:<br>Given the reports and the published articles, the exclusion of Rebif does not negatively impact clinical outcomes of the patients.<br>Level of evidence: clinical experts' opinion	Like Avonex:<br>Few small share of patients who used this interferon received Rebif.<br>Clinical experts indicate that those patients could shift to the biosimilars without occurring clinical hazard. If they shift, OOP will decrease.<br>Level of evidence: real world evidence ((secretariat for HCHI)<br>and clinical experts' opinion	Estimated saving for social health insurance agencies: 147,882 US$ annually<br>Level of evidence: real world evidence (secretariat for HCHI and Social health insurance agencies)

Table S1: Performance of selected MS services collected by TFEC in step D2.

Service	Quality (clinical impact: effectiveness and safety)	Necessity (OOP and alternative availability)	Sustainability (Budget impact)
Actovex/Cinnovex	Given the reports and the published articles, the implementation of the policy of internal reference pricing may not cause a significant change in the quality of the service offered. Although there is no comparative study, no clinically or statistically significant difference has been observed between Actovex and Cinnovex in RRMS patients.<br>Clinical experts indicate Actovex is not formulated with albumin-free intermediate materials in comparison with Cinnovex. So this may cause some clinical inconveniences for patients who received Actovex. But IFDA formally rejected this clinical judgement.<br>Level of evidence: clinical experts' opinion	The implementation of this policy will increase patients' out of pocket payment which is influenced by the rate of the shift of the patients from Cinnovex to Actovex.<br>Level of evidence: real world evidence ((secretariat for HCHI) and payer experts' opinion	Estimated saving for social health insurance agencies: 6,590,595 US$ annually<br>Level of evidence: real world evidence (secretariat for HCHI and Social health insurance agencies)
