# Supplementary material for: The Use of Evidence-Informed Deliberative Processes for Health Insurance Benefit Package Revision in Iran
Source: Int J Health Policy Manag. 2022 Mar 2;11(11):2719–26. doi: 10.34172/ijhpm.2022.6485 (PMC9818091; doi:10.34172/ijhpm.2022.6485)
Supplement: Supplementary file 2 — Evidence on Multiple Sclerosis and Available Services for Cluster 1 and 2 (as Collected by TFEC in Step C). [file ijhpm-11-2719-s002.pdf]

Article title: Journal name: International Journal of Health Policy and Management (IJHPM)

Authors' information: Mojtaba Nouhi[1], Rob Baltussen[2]*, Seyed Sajad Razavi[3], Leon Bijlmakers[2], Moahmmad Ali Sahraian[4], Zahra Goudarzi[5], Parisa Farokhian[6], Jamaledding Khedmati[7], Reza Jahangiri[8], Alireza Olyaeemanesh[9]*

[1]National Institute for Health Research, Tehran University of Medical Sciences,Tehran, Iran.

[2]Radboud University Medical Centre, Nijmegen, The Netherlands.

[3]Mofid Children Hospital, Shahid Beheshti University of Medical Sciences, Tehran, Iran.

[4]Multiple Sclerosis Research Center, Neuroscience Institute, Tehran University of Medical Sciences, Tehran, Iran.

[5]Health Human Resources Research Center, Department of Health Economics, School of Management and Medical Informatics, Shiraz University of Medical Sciences, Shiraz, Iran.

[6]High Council for Health Insurance, Ministry of Health and Medical Education, Tehran, Iran.

[7]Pharmaceutical Management and Economics Research Center, Tehran University of Medical Sciences, Tehran, Iran.

[8]Health Management and Economics Research Center, Iran University of Medical Sciences, Tehran, Iran.

[9]Health Equity Research Center, Tehran University of Medical Sciences, Tehran, Iran.

(*Corresponding author: Email: rob.baltussen@radboudumc.nl & arolyaee@gmail.com)

Supplementary file 2. Evidence on Multiple Sclerosis and Available Services for Cluster 1 and 2 (as Collected by TFEC in Step C)

This annex describes the collection of basic evidence on a range of MS services, divided into five clusters. It served as an input to the TWG meeting on scoping to identify aspects that required closer evaluation, and/or additional evidence. The five service clusters, based on the natural history of MS, are: Diagnosis and risk stratification, Clinical management, Symptom management, Relapse management, and Follow-up. The evidence is extracted from the database of the social security organisation, which provides services to some 60% of the MS patients, from March 2019 to March 2020.

Cluster 1: Diagnosis and Risk Stratification

Epidemiology

The local epidemiological surveys show that the incidence of new MS cases in Iran, regardless of gender difference, is 3.2 per 100000 individuals, which culminates to a total 2856 cases taking into account the Iran's population which is 84 billion people. However, the number of new cases recorded by MSPSU has been 4200 cases in 2019. Examining medicine registration systems at SSO revealed that in 2019 about 5412 new MS patients have registered, which reflects a 10 percent increase in the number of new patients.

New cases detected by SSO were diversely distributed through the country. Tehran, Alborz and Isfahan had observed more new cases, reporting 12 to 14 cases per 100000 people. Fars, East Azarbaijan, Yazd, and Semnan provinces reported six to nine cases per 100000 people. Most had reported four to five new cases per 100000 people including Guilan, Qom, Markazi, Boushehr, Hamedan, Qazvin, Ardabil, Razavi Khorasan, Kerman, Mazandaran, Chahar Mahal va Bakhtiari and Kermanshah. The rest of the provinces -Ilam, Zanjan, Kordestan, Khuzestan, West Azerbaijan, Kohgiluyeh va Boyer Ahmad, Golestan, Lorestan, Hormozgan, Northern Khorasan, Sistan va Balochestan- had reported up to four cases per 100000 people. The figure S1 demonstrates the distribution of new MS cases in 2019 as provided by SSO.

There has been much debate regarding the reason for such diverse distribution across different provinces. The economic development of the province, people's lifestyle, heredity and access to health care and diagnosis services are considered as some underlying reasons.

Service use

The diagnosis of MS may make it difficult to differentiate it from other similar diseases. The available evidence indicates that in 2019 more than 400 different codes of laboratory diagnosis and imaging services have been used to clinically diagnose patients suffering from

MS. The average number of diagnosis services prescribed for suspected patients in the first visits was 24 cases.

One of the most important services utilized for the diagnosis of MS is MRI; therefore, determining the frequency of access to this service is critical. Available evidence shows that 15 percent of the newly diagnosed patients have had access to MRI once a year, 46 percent of them have utilized MRI twice a year and the remaining cases have utilized MRI more than twice a year. The details of access to this service are presented in the figure S1.

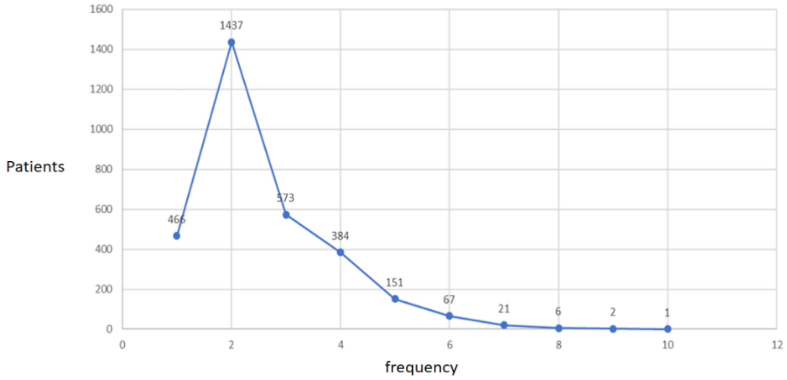

Figure S1. MRI utilization in patients with MS

Another factor reflecting the quality of care in the diagnosis and risk stratification cluster is to pinpoint what has been provided as the first service immediately after the patient is diagnosed with MS. In this regard, the data suggests that the services offered to the patients after diagnosis and determining the risk has been Disease Modifying Therapies (DMT), in a way that 93 percent of patients received one of the DMTs and only seven percent of them did not. Among those who utilized DMTs, about 33 percent had received brand-name or locally produced Interferons, 18 percent had received Dimethyl fumerate, 15.5 percent had received Rituximab, 12.5 percent had received Glatiramer acetate and 7.8 percent had received Fingolimod. More details are provided in the figure S2.

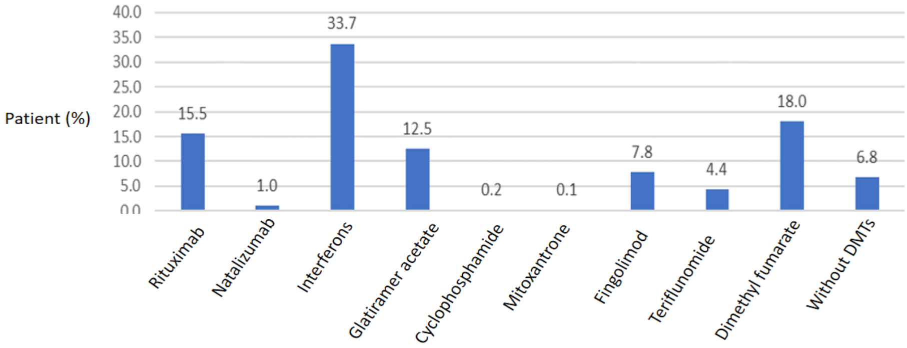

Figure S1. The first intervention for newly-diagnosed patients

As depicted in the figure above, very few newly diagnosed patients received Cyclophosphamide, Mitoxantrone, and Natalizumab as their first treatment.

Coverage by HIBP and out-of pocket costs

Most services currently offered to patients in order to diagnose and determine the risk of MS are covered by social health insurance agencies. If patients refer to the private sector for laboratory and imaging services, they have to pay not only 30 percent of the service cost, but also the differential amount between public and private sectors' tariffs.

Since most of the patients refer to private sectors for outpatient laboratory and imaging services, their cost sharing can increase. The average cost paid by suspected patients or newly diagnosed ones in 2019 related the diagnosis services cost sharing has increased up to US$ 26.6, to which the differential amount between the public and private tariffs should be added.

Costs and budget impact

In terms of the cost and financial factor, the data provided by SSO for the year 2019 indicates that US$ 342,857 spent on new MS cases have been used for differential diagnosis and determining the risk. However, 20 high frequency services prescribed for patients comprised 60 percent of the total cost. The following table S1 provides the details related to the high frequency services and the cost related to the services.

Table S1. Laboratory and imaging diagnosis services for newly-diagnosed patients

	Service	Frequency	Insurance costs ($US)
1	Cell Blood Count (CBC) and WBC differential	13,664	8,371
2	ALT SGPT in serum / plasma	11,372	5,180
3	AST SGOT in serum / plasma	11,337	5,163
4	Intravenous or capillary blood injection once or more using vacuum tube	10,188	5,434
5	Quantitative test of Creatinine in blood / serum / plasma creatinine	6,135	2,259
6	Intravenous or capillary blood draw, once or more	6,127	1,683
7	Quantitative test of urea in blood /serum / plasma	5,905	1,738
8	Quantitative test of Alkaline Phosphatase Activity (ALP) in serum / plasma	5,209	2,375

Table S1. Laboratory and imaging diagnosis services for newly-diagnosed patients

	Service	Frequency	Insurance costs ($US)
9	Quantitative test of Thyroid stimulating hormone serum / plasma	5,143	7,645
10	Complete urine test	4,516	1,573
11	Quantitative test of glucose in blood / serum / plasma	4,312	1,427
12	Erythrocyte sedimentation rate test (ESR)	4,144	821
13	Quantitative test of Hydroxy Vitamin D-25 in serum / Plasma	3,418	6,295
14	Quantitative test of Triglyceride (TG) in blood / serum / plasma	3,002	1,476
15	Quantitative test of cholesterol in blood / serum / plasma	2,991	1,093
16	Qualitative / semi-quantitative CRP in serum / plasma	2,659	910
17	Quantitative test of T4 in serum /plasma	2,537	2,920
18	Quantitative test of HDL-Cholesterol in serum / plasma	2,321	1,104
19	Qualitative or semi-quantitative test of ANA (Anti – Nuclear Antibody) using immunofluorescence method	2,215	5,758
20	HBsAg test	2,138	6,914

Cluster 2: Clinical Management

The various Disease Modifying Therapies (called DMTs) are described in this cluster. The commonly prescribed intervention for MS patients is prescribing medicines. Different lines of treatment are analyzed below.

Service use

In the clinical practice guidelines for MS, the approved DMTs used in the treatment process of MS patients are elaborated.

Therapeutic medicines for MS include a variety of Interferons, Teriflunomide, Natalizumab, Fingolimod, Glatiramer acetate, Dimethyl fumarate, Ocrelizumab, Alemtuzumab and Mitoxantrone. The patients will receive one of the DMTs depending on the progression of the disease, type of the disease, previous medications received and other clinical factors. It should be noted that the patients' acceptance and financial affordance of the medicine along

with other non-clinical motivations of the physician prescribing the medicine are of prime importance.

A factor that can be an indicator of the quality of service provided is patients' shift to other DMTs that might be due to the disease progression or other patient-related factors. This shift in medication along with the accompanied influences and important symptoms for analysis of the patient's health status, can also reflect the change in how physicians prescribe and recommend the medicines.

The findings suggest that over the last year significant changes have occurred in the medicine access, and shifts to new medicines have been observed. More than 25 percent of patients who received Natalizumab shifted to Rituximab which is a medicine used for next level treatment line. The figure S3 exhibits more findings.

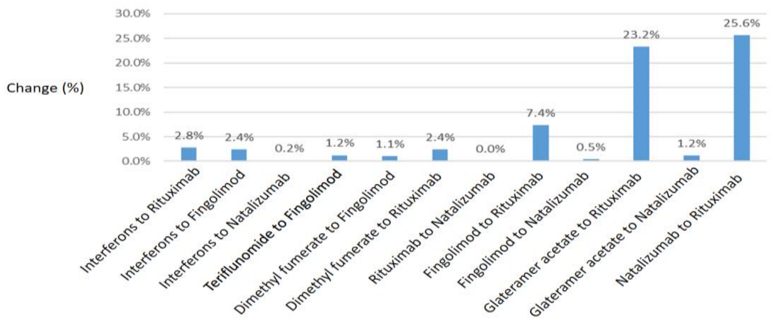

Figure S3. Main switches between DMTs

As evident in the figure S3, more than 23 percent of patients receiving Glatiramer acetate as their base therapy have shifted to Rituximab. This shift most probably reflects the progression of the disease in this group of patients as Glatiramer acetate is recognized as a first-line treatment and Rituximab as a next level treatment line. A shift from Fingolimod to Rituximab is another case of such shift.

The results showed that more than seven percent of patients who received Fingolimod shifted to Rituximab which can be a sign of disease progression in these patients. In other words, Natalizumab, Glatiramer acetate, and Fingolimod are the three medicines that had witnessed the most shifts to other medicines relative to the total number of patients.

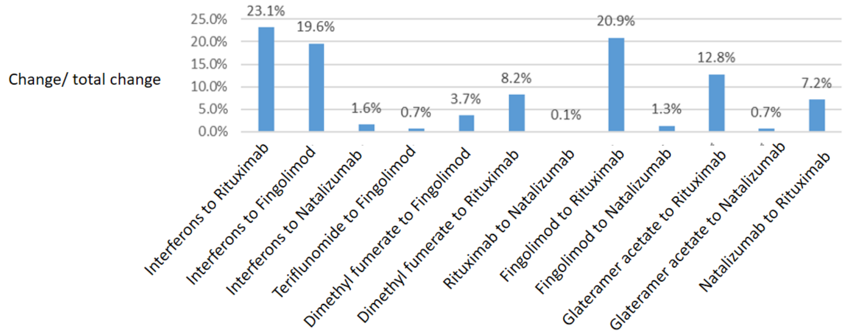

Figure S4. The share of main switches of DMTs from total switches

Another way to showcase the shifts in DMTs is measuring what the total shifts occurred in a year has been and which one has been the most frequent. The results revealed that 23 percent of patients who received one type of interferon had shifted to other medicines. In other words, 23 percent of the shifts has been a shift from one type of Interferon to another DMT. Another case encompassing nearly 21 percent of the total shifts was a shift from Fingolimod to Rituximab. A shift from Interferon to Fingolimod was another instance, ranking third with a total of 19 percent of the total number of shifts. Still, another instance was a shift from Glatiramer acetate to Rituximab showing a 13 percent of the total shifts. The figure S4 exhibits the details of other shifts.

The difference in the extent of access to services among different patients, which is called practice variation in literature, can be a reflection of the quality of service delivery. An overview of practice variation can provide valuable information such as the diversity in the number of prescribed medicines per patient, the number of patient per physician and the prescription techniques used. For instance, in case of Natalizumab, it was observed that in 2019 only 29 out of 680 patients had received the medicine properly; that is, based on the clinical practice guidelines. The rest of the patients had received it in different doses as evident in the figure S5.

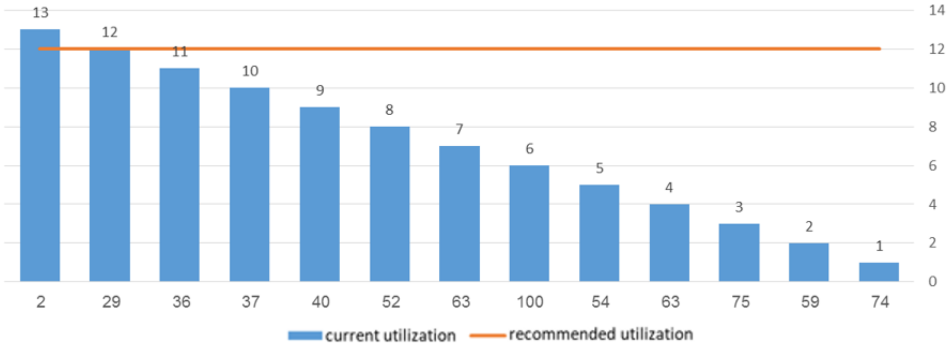

Figure S5. The practice variation of Natalizumab

However, recent modifications in clinical practice guidelines of MS allows receiving Natalizumab at an interval of every one to two months. Considering all these issues, Natalizumab consumption has witnessed a significant amount of variation.

The consumption of Fingolimod is another notable case. The studies show that the there is a large variation regarding the access to this medicine. The figure S6 shows the imbalanced distribution regarding patients' access to this medicine.

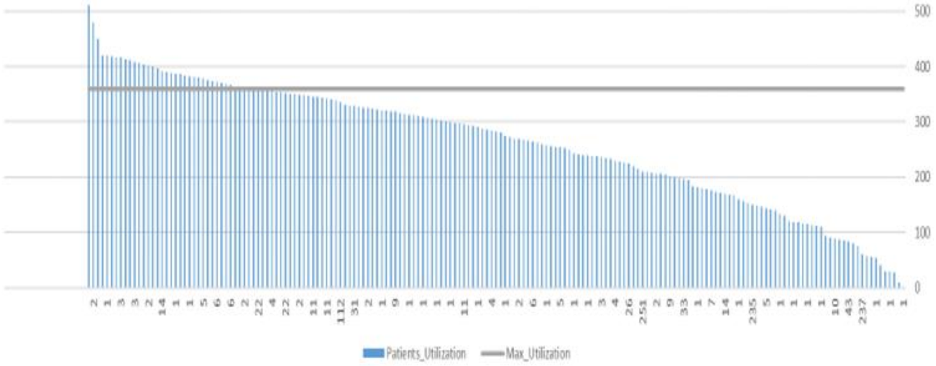

Figure S6. The practice variation of Fingolimod

HIBP coverage and out-of-pocket payment

The amount of cost sharing and the total cost sharing is different for each of the medicines mentioned in this cluster. The price of different DMTs is different leading to a difference in the amount of OOP. Since most patients have to receive these medicines, it is vital to provide financial support for these types of medicines.

To exemplify, patient cost sharing related to Natalizumab -as one of the most expensive DMTs- is ten percent of consumer price. Patient cost sharing is five percent of the medicine price for Fingolimod and 13 percent for Rituximab, on average. A huge share of annual cost sharing is afforded by patients receiving Natalizumab, followed by Teriflunomide and Interferon respectively. The figure S7 presents the details of the out of pocket payment for patients in 2019.

The colors used in the figure S7 represents the total cost of the three criteria, namely the cost per patient, OOP and the number of patients. Interferon was the most frequently used DMTs in 2019. Mitoxantrone (96 patients), Cyclophosphamide (180 patients) and Natalizumab (683 patients) were the medicines with the smallest number of the patients, respectively.

A notable fact is that the OOP has reduced, resulting in provision of a better financial support for the patients, thanks to the government's plan for some targeted disease such as MS, Thalassemia and Hemophilia.

Cost and budget impact

In order to assess the sustainability of DMTs, different indicators were utilized, including the prices of medicine, the number of medicines prescribed, the insurance cost per patient, the total cost per medicine, the total insurance cost per medicine and the number of patients receiving at least one dose for each medicine type.

Regarding the price of the medicine, it was observed that Natalizumab is the most expensive DMTs per dose. The next is Rituximab, being the most expensive per unit in 2019.

Considering the number of medicines consumed, the findings showed that Dimethyl fumerate, Fingolimod and Interferon had witnessed the most consumption, respectively .

The insurance cost per patient was another indicator. As the studies revealed, Natalizumab had the most annual insurance cost for the insurers, followed by Fingolimod.

However, the highest total insurance cost belonged to the Interferons which could be traced to the large number of patients receiving them. The figure S7 presents more details in this regard.

medicie	price	frequency	insurance cost per patient	total cost	total insurance cost	out of pocket	cost sharing (%)	size of the patients
Natalizumab	1528	3921	7908	6001673	5401352	878	10	683
Interferon	33	1118626	1478	34192153	29587210	230	13	20018
Rituximab	544	24910	1371	13470799	12123719	152	10	8843
Dimethylfumerate	2	2446468	498	5895661	4128405	213	30	8282
Fingolimod	11	1788217	2671	19360069	18392529	141	5	6886
Glatiramer Acetate	29	73732	1434	2125593	1913249	159	10	1334
Cyclophosphomide	7	1032	32	6374	5735	4	10	180
Triflunomide	10	279189	1297	2725114	1910731	554	30	1472
Mitoxantron	47	190	83	8889	8000	9	10	96

Figure S7. Utilization and budget impact (US$) of DMTs

As represented in the figure S7, the number of patients receiving at least one unit of Interferons is much more in comparison with the number of patients receiving other medicine types. It should be noted that all DMTs have covered different patient numbers and insurance costs. For instance, nearly 42 percent of the patients in 2019 have received at least one unit of Interferons which encompasses more than 40 percent of the total insurance cost of DMTs.

It was also found that while slightly more than 25 percent of the total insurance cost of DMTs was related to Fingolimod, only 14 percent of patients had received this medicine.

The analyses revealed that 16.5 percent of DMTs costs have been due to Rituximab, with 17 percent of total patients receiving DMTs having received at least one dose of it. Natalizumab

included more than seven percent of the DMTs costs while only 1.4 percent of the patients had received it. The details on the distribution costs and the number of patients receiving different DMTs are presented in the figure S8.

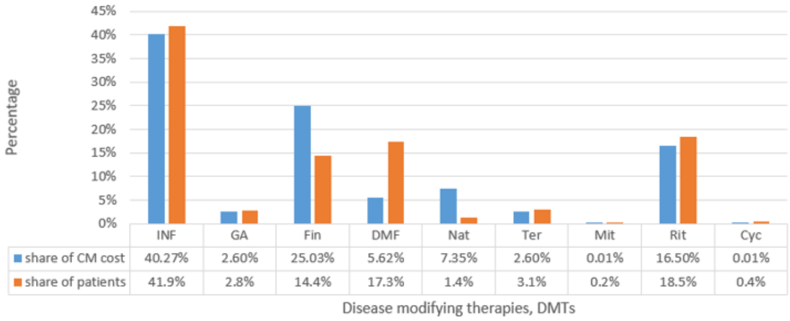

Figure S8. The share of cost(US$) and the number of patients across different DMTs

Regarding the distribution of patients and DMTs costs across different treatment lines, findings suggest that about 65 percent of the patients had received first line treatment, encompassing 51 percent of the total DMTs costs. Besides, 16 percent of the patients received second-line medicines which covered about 32 percent of the total cost. As for the third line, 19 percent of patients were present and it covered 17 percent of the total costs .This distribution can be seen in the Figure S9.

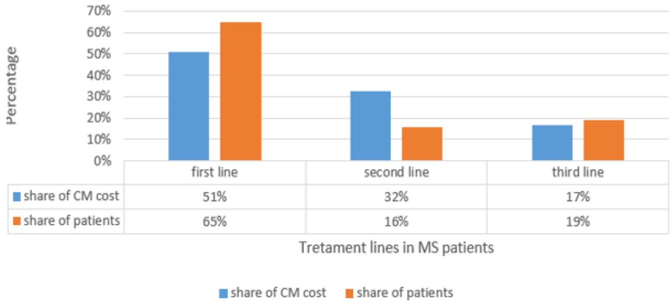

Figure S9. Utilization and budget impact (US$) of DMTs in treatment lines
