# Supplementary material for: The Use of Evidence-Informed Deliberative Processes for Health Insurance Benefit Package Revision in Iran
Source: Int J Health Policy Manag. 2022 Mar 2;11(11):2719–26. doi: 10.34172/ijhpm.2022.6485 (PMC9818091; doi:10.34172/ijhpm.2022.6485)
Supplement: Supplementary file 3 — Evidence on Multiple Sclerosis and Available Services for Cluster 3-5 (as Collected by TFEC in Step C). [file ijhpm-11-2719-s003.pdf]

Article title: Journal name: International Journal of Health Policy and Management (IJHPM)

Authors' information: Mojtaba Nouhi[1], Rob Baltussen[2]*, Seyed Sajad Razavi[3], Leon Bijlmakers[2], Moahmmad Ali Sahraian[4], Zahra Goudarzi[5], Parisa Farokhian[6], Jamaledding Khedmati[7], Reza Jahangiri[8], Alireza Olyaeemanesh[9]*

[1]National Institute for Health Research, Tehran University of Medical Sciences,Tehran, Iran.

[2]Radboud University Medical Centre, Nijmegen, The Netherlands.

[3]Mofid Children Hospital, Shahid Beheshti University of Medical Sciences, Tehran, Iran.

[4]Multiple Sclerosis Research Center, Neuroscience Institute, Tehran University of Medical Sciences, Tehran, Iran.

[5]Health Human Resources Research Center, Department of Health Economics, School of Management and Medical Informatics, Shiraz University of Medical Sciences, Shiraz, Iran.

[6]High Council for Health Insurance, Ministry of Health and Medical Education, Tehran, Iran.

[7]Pharmaceutical Management and Economics Research Center, Tehran University of Medical Sciences, Tehran, Iran.

[8]Health Management and Economics Research Center, Iran University of Medical Sciences, Tehran, Iran.

[9]Health Equity Research Center, Tehran University of Medical Sciences, Tehran, Iran.

(*Corresponding author: Email: rob.baltussen@radboudumc.nl & arolyaee@gmail.com)

Supplementary file 3. Evidence on Multiple Sclerosis and Available Services for Cluster 3-5 (as Collected by TFEC in Step C)

Cluster 3: Symptom Management

The services used for managing symptoms in MS patients are analyzed below.

Service use

577 different medicines were used to manage clinical complications of MS and also treatment-associated morbidities. More than 48,000 patients (48,488) had received at least one of the services in relapse management cluster. These services were used with varying frequency.
One reason for such variation in managing symptom could be that clinical practice guidelines for MS do not explicitly provide evidence-based recommendations for managing common symptoms such as depression, urinary incontinence, fatigue, and mobility malfunctions. The thirty high frequency laboratory and imaging services are presented in the table S1.

Table S1. Medication used by the patients in MS symptom management

Number	Medicine name	Frequency
1	FAMPRIDINE 10MG ER TAB - - TABLET	1,759,150
2	GABAPENTIN 100 CAP - - CAPSULE	1,159,818
3	AMANTADINE HCL 100MG CAP - 100MG - CAPSULE	1,056,531
4	BACLOFEN 25MG TAB - 25MG - TABLET	1,033,214
5	GABAPENTIN 300 CAP - - CAPSULE	747,965
6	BACLOFEN 10MG TAB - 10MG - TABLET	659,283
7	VITAMIN B1 (THIAMINE HCL)	568,411
8	HALOTHANE NICHOLAS PIRAMAL 250ML	546,000
9	SERTRALIN HCL 50MG TAB - 50MG - TABLET	519,173
10	CALCIUM-D TAB - - TABLET	468,786
11	PROPRANOLOL HCL 10 TAB - - TABLET	454,989
12	FLUOXETINE HCL 20MG CAP - 20MG - CAPSULE	426,493
13	LEVOTHYROXINE SODIUM 0.1MG TAB	398,833
14	FOLIC ACID 1MG TAB - 1MG - TABLET	367,678
15	AZATHIOPRINE 50MG TAB - 50MG - TABLET	355,973

Table S1. Medication used by the patients in MS symptom management

Number	Medicine name	Frequency
16	VITAMIN D3 (COLECALCIFEROL) PEARL - - PEARL	342,378
17	CARBAMAZEPINE 200MG TAB - 200MG - TABLET	336,951
18	PROPRANOLOL HCL 20MG TAB - - TABLET	336,827
19	ASA 80MG EC TAB - 80MG – TABLET	306,738
20	CITALOPRAM HBR 20MG FC TAB - 20MG - TABLET	304,899
21	FERROUS SULFATE-F TAB - - TABLET	271,812
22	RANITIDINE 150MG TAB - 150MG - TABLET	243,008
23	CHLORDIAZEPOXIDE 5MG TAB - 5MG - TABLET	237,069
24	VALPROATE SODIUM SR 500MG TAB - 500MG - TABLET	234,406
25	ATORVASTATIN 20MG TAB - 20MG - TABLET	222,319
26	ALPRAZOLAM 0.5MG TAB - 0.5MG - TABLET	216,849
27	CLONAZEPAM 1MG TAB - 1MG – TABLET	207,712
28	NORTRIPTYLINE HCL 10MG TAB - - TABLET	200,059
29	VALPROATE SODIUM 200MG TAB - 200MG - TABLET	198,827
30	PREDNISOLONE 5MG TAB - 5MG – TABLET	179,296

HIBP coverage and out-of-pocket payment

Services needed to treat the complications of MS were covered by HIBP. Treatment of complications of MS is provided on an outpatient platform and 70 % of the service price is paid by social health insurance agencies. Therefore, the out-of-pocket payment was 30% of the service price.

Cost and budget impact

The 50 items with the highest frequency accounted for 43% of the total cost. SSO had spent 4,523,809 million US$ on symptom management cluster. Cost per each patient was 76US$ in symptom management cluster, which is lower than the cost per patient in clinical management cluster.

Cluster four: Relapse management

In this cluster, the services related to treating relapse in patients with MS are analyzed.

Service use

Despite the limited local information on the number of relapse in patients with MS, it was estimated that about half of the patients receiving DMTs had one seizure per year (probability = 0.49)[1]. The severity of the relapse can range from mild to more severe, requiring long-term hospitalization and more complex medical care. The clinical practice guidelines for MS recommends three different levels of treatment for patients with relapse, including pulse therapy with prednisolone, plasma therapy using albumin, and Intravenous immunoglobulin (IVIG).

HIBP coverage and out-of-pocket payment

Unlike other MS services, most of the services available in the relapse management cluster are provided in a hospital setting, i.e. inpatient platform. Not only the core services of relapse management that recommended in clinical practice guidelines for MS, but any other diagnostic and treatment services performed for these patients in hospital are covered by HIBP. Generally, the coverage of these services is 90% of the cost, the rest (10 %) is paid by the patients.

Cost and budget impact

The average cost that SSO paid for each patient in the relapse management box was $ 516. The total cost paid by SSO in this cluster was 7,380,952 US$.

Cluster five: Follow-up

In this cluster, the services related to follow-up of MS patients are analyzed.

Service use

A total of 625 different follow-up services were used by patients in 2019. More than 42,000 of MS patients (42,222 cases), encompassing about 81% of the total SSO patients, have used the follow-up services. This means that 19% of the patients have not received their follow-up

---

[1] Inusah S, Sormani MP, Cofield SS, et al. Assessing changes in relapse rates in multiple sclerosis. Multiple Sclerosis Journal. 2010;16(12):1414-1421. doi:10.1177/1352458510379246.

services, due to several reasons. The thirty high frequency laboratory and imaging services are presented in the table S2.

Table S2. The thirty most frequent services used in follow-up cluster

	Services	Frequency	Insurance costs (US$)
1	CBC test	72,991	44,668
2	ALT SGPT test in serum / plasma	61,990	28,064
3	AST SGPT test in serum / plasma	61,618	27,895
4	Intravenous or capillary blood injection once or more using vacuum tube	51,077	27,190
5	Quantitative test of Creatinine in blood / serum / plasma	35,265	12,955
6	Quantitative test of urea in blood /serum / plasma	33,698	9,898
7	Quantitative test of Thyroid stimulating hormone serum / plasma	28,561	42,365
8	Intravenous or capillary blood injection once or more	28,485	7,749
9	Complete urine culture using tape or tablets	28,415	9,895
10	Quantitative test of Hydroxy Vitamin D-25 in serum / Plasma	27,109	50,063
11	Quantitative test of Alkaline Phosphatase Activity (ALP) in serum / plasma	26,962	12,276
12	Quantitative test of blood glucose in serum / plasma	25,771	8,468
13	Quantitative test of Triglycerides in blood / serum / plasma	18,327	9,008
14	Quantitative test of Cholesterol serum / plasma	18,273	6,676
15	Erythrocyte sedimentation rate test (ESR)	17,367	3,449

Table S2. The thirty most frequent services used in follow-up cluster

	Services	Frequency	Insurance costs (US$)
16	Brain MRI with and without contrast agent	16,961	452,585
17	Complete semen analysis	16,374	12,355
18	Quantitative test of HDL-Cholesterol	14,477	16,138
19	Quantitative test of T4 in serum	14,053	9,110
20	Quantitative test of Bilirubin in serum / plasma	12,696	327,586
21	Cervical MRI with or without contrast agent	12,238	5,087
22	Quantitative test of Calcium in serum / plasma	11,187	11,876
23	Quantitative test of T3 in serum / plasma	10,351	5,344
24	Quantitative test of LDL-Cholesterol	10,103	29,354
25	Quantitative test of HBsAg	9,052	3,400
26	Quantitative test of Phosphorus in serum / plasma	8,863	2,991
27	Quantitative / semi-quantitative CRP in serum	8,700	21,921
28	Quantitative test of Ferritin in serum / plasma	470 ,	21,921
29	Quantitative test of Potassium in blood / serum / plasma	7,688	3,278
30	Quantitative test of Sodium in blood /plasma / serum	7,539	3,206

A crucial issue in the follow-up cluster was the fact that the clinical practice guidelines on how to keep track of patient services is not transparent and needs to be amended and supplemented.

HIBP coverage and out-of-pocket payment

Patients have access to all the required services in the follow-up box and these services are covered by the insurance organizations. All Services in the follow-up cluster are provided to patients on an outpatient platform. The share of the patient in the services is 30% of the price of each service because according to the regulations of social health insurance agencies, the services that are provided on an outpatient platform have up to 70% insurance coverage.

Cost and budget impact

60% of the follow-up costs are related to services with highest frequency (Table S2).
The total cost of 2,595,238 US$ paid by SSO and the share of patients was 761,904 US$.
The average cost per patient on insurance perspective was 62 US$ and the average amount of out-of-pocket payment per patient was 18 US$.
